# Impacts of Indoor Dust Exposure on Human Colonic Cell Viability, Cytotoxicity and Apoptosis

**DOI:** 10.3390/toxics11070633

**Published:** 2023-07-21

**Authors:** Noura Abdulrahman, Trenton J. Honda, Ayat Ali, Nabras Abdulrahman, Daniel Vrinceanu, Shishir Shishodia

**Affiliations:** 1 Department of Environmental and Interdisciplinary Sciences, Texas Southern University, Houston, TX 77004, USA; n.abdulrahman4473@student.tsu.edu (N.A.);; 2 School of Clinical and Rehabilitation Sciences, Northeastern University, Boston, MA 02115, USA; 3 Department of Physics, Texas Southern University, Houston, TX 77004, USA

**Keywords:** trace metal dust, organic contaminant dust, indoor dust, viability, cytotoxicity, apoptosis

## Abstract

Introduction: Environmental exposure to indoor dust is known to be associated with myriad health conditions, especially among children. Established routes of exposure include inhalation and non-dietary ingestion, which result in the direct exposure of gastrointestinal epithelia to indoor dust. Despite this, little prior research is available on the impacts of indoor dust on the health of human gastrointestinal tissue. Methods: Cultured human colonic (CCD841) cells were exposed for 24 h to standard trace metal dust (TMD) and organic contaminant dust (OD) samples at the following concentrations: 0, 10, 25, 50, 75, 100, 250, and 500 µg/mL. Cell viability was assessed using an MTT assay and protease analysis (glycyl-phenylalanyl-aminofluorocoumarin (GF-AFC)); cytotoxicity was assessed with a lactate dehydrogenase release assay, and apoptosis was assessed using a Caspase-Glo 3/7 activation assay. Results: TMD and OD decreased cellular metabolic and protease activity and increased apoptosis and biomarkers of cell membrane damage (LDH) in CCD841 human colonic epithelial cells. Patterns appeared to be, in general, dose-dependent, with the highest TMD and OD exposures associated with the largest increases in apoptosis and LDH, as well as with the largest decrements in metabolic and protease activities. Conclusions: TMD and OD exposure were associated with markers of reduced viability and increased cytotoxicity and apoptosis in human colonic cells. These findings add important information to the understanding of the physiologic effects of indoor dust exposure on human health. The doses used in our study represent a range of potential exposure levels, and the effects observed at the higher doses may not necessarily occur under typical exposure conditions. The effects of long-term, low-dose exposure to indoor dust are still not fully understood and warrant further investigation. Future research should explore these physiological mechanisms to further our understanding and inform public health interventions.

## 1. Introduction

Indoor dust is a ubiquitous environmental exposure that has been linked to detrimental health outcomes, especially among children [1,2,3,4]. Indoor dust is composed of semi-volatile organic compounds, heavy metals, and allergens that can enter the human body not only by the inhalation of re-suspended and suspended particles, but also via the non-dietary ingestion of dust particles adhering to objects, the skin, and food, including direct absorption through the skin [4,5].

Sources of indoor dust include: insects, food particles, fibers, bacteria, smoke, and heavy metals [6]. The organic and inorganic chemicals and toxicants that make up indoor dust vary widely, and contribute to a broad swath of perturbations in health [7]. Trace metals found in dust, such as lead, cadmium, and arsenic, can have toxic effects on human health. Organic contaminants, including polychlorinated biphenyls (PCBs), polybrominated diphenyl ethers (PBDEs), and phthalates, have been associated with a range of health effects, including endocrine disruption and carcinogenicity [8,9,10,11].

Respiratory issues, eye problems, throat complications, and cancers have all been linked to indoor dust [3,12]. Aerosolized metals, which are common components of indoor dust, have also been previously associated with impaired neurological integrity in children, morbidity and mortality in adults from several causes, lead poisoning, and allergic reactions [13,14,15,16].

While much research has been conducted on the effects of inhaled dust, less is known about the impacts of ingested dust on general health, and on gastrointestinal health in particular. The gastrointestinal tract is the first point of contact for ingested dust and its contaminants. Therefore, understanding the effects of dust ingestion on cells representative of the gastrointestinal tract is crucial. This study aims to investigate the effects of standard samples of indoor dust on the viability of normal human cells representative of the colon (CCD 841 cells).

Children are more vulnerable to the potential effects of indoor environmental contaminants such as dust for both physiological and behavioral reasons. Physiologically, children have a higher minute ventilation than adults, a propensity for mouth breathing, which allows a greater volume of pollutants to be inhaled and diminishes nasal filtration of particles [17,18], and incompletely developed immune and respiratory systems [17]. Children are also shorter and often play at ground level, both of which can lead to exposure to higher doses of pollutants that are denser than air and thus are in a higher concentration closer to the ground [19]. Children also have a greater propensity for hand–mouth behaviors than adults, which provides a larger level of gastrointestinal exposure to potential environmental contaminants [20]. Much of the previous research has linked childhood environmental exposures to adverse health outcomes. For example, particulate matter (PM) in the air has been linked to asthma—a disease that has been on the rise for the last 20 years [21,22,23,24]—as well as an increased incidence of childhood respiratory infections [25,26], cancer [27,28,29], and developmental and behavioral issues [30,31], while indoor dust exposure has also been linked to cancers and gastrointestinal disorders [3,32].

While gastrointestinal intake is a known primary route of indoor dust exposure, few prior studies have investigated the specific effects of different indoor dust contaminants on gastrointestinal cytotoxicity and cell viability. The evaluation of cell viability is essential for determining the effectiveness of a novel medicine or treatment, or the effects of a component, which is in this case pollutants. Cell viability is an assessment of a cell’s health in general. Agents operating on cells, such as contaminants, have varying degrees of influence on the cell’s survival and functioning. The vitality of cells is a good indicator of how they will react to pollutants [33].

To address these gaps in the current literature, we examined the effects of dust samples on normal human colon (CCD841) cell viability and cytotoxicity. We hypothesized that normal human colon cells exposed to dust particles will have reduced viability and increased cytotoxicity and apoptosis.

## 2. Methods

### 2.1. Reagents

Eagle’s Minimum Essential Medium (EMEM), Fetal Bovine Serum (FBS), Phosphate-Buffered Saline (PBS), and Antibiotic–Antimycotic (Anti-Anti) were purchased from Gibco Life Technologies (Gaithersburg, MD, USA). Dimethyl Sulfoxide (DMSO) was purchased from Thermo Fisher Scientific (Waltham, MA, USA).

### 2.2. Indoor Dust Samples and Preparation

Standard samples of trace metal dust (TMD) and organic contaminant dust (OD) from indoor dust were purchased from the National Institute of Standards and Technology (NIST, Gaithersburg, MD, USA) [34,35]. The TMD sample is a composite mixture that represents a variety of trace metals commonly found in indoor dust, including but not limited to iron, lead, and zinc. On the other hand, the OD sample encompasses a wide spectrum of organic pollutants frequently present in indoor dust, such as polychlorinated biphenyls (PCBs), polyaromatic hydrocarbons (PAHs), and phthalates. These samples were prepared from bulk dust collected from a multitude of residential sites across the United States, ensuring a representative composition that reflects typical indoor dust contaminants. It is important to note that while these samples are standardized, minor variations may occur between different batches. To mitigate the potential impact of these variations, all experimental procedures were conducted using dust samples procured from the same batch.

For the preparation, 50 mg of each dust type was diluted in 1 mL Phosphate-Buffered Saline (PBS), vortexed, transferred to a dark glass vial, and finally stored at −80 °C. Dust sample concentrations of 0, 10, 25, 50, 75, 100, 250, and 500 µg/mL were prepared in 5 mL of a complete media in eight separate 25 mL tubes. After use, these concentrations were refrigerated at −20 °C for future use.

### 2.3. Cell Culture of CCD841

After receiving the CCD841 CoN cells (ATCC^®^ CRL-1790™), which were of female embryonic origin (21 weeks gestation), cells were cultured in Eagle’s Minimum Essential Medium (EMEM), which was supplemented with 5% Fetal Bovine Serum (FBS), 0.5% EGF, and 1% Antibiotic–Antimycotic, and grown in a cell culture incubator at 37 °C for 24 h before passaging.

### 2.4. Experimental Design

Healthy human colon epithelial cells (CCD841) were exposed to two different forms of indoor dust, which were categorized based on their chemical contents: trace metal dust (TMD) and organic contaminant dust (OD). An MTT test and glycyl-phenylalanyl-aminofluorocoumarin (GF-AFC) analyses were used to examine cell viability 24 h following exposure to dust at concentrations of 0, 10, 25, 50, 75, 100, 250, and 500 μg/mL.

For each analysis (i.e., MTT viability, GF-AFC viability, caspase 3/7 apoptosis, and LDH-GLO cytotoxicity), microplate treatments were applied on 4 independent 96-well plates with triplicates of each sample. In addition, three negative controls were included, which consisted of wells containing the cell culture medium alone. In this context, the ‘negative control’ refers to a condition that is expected to show no effect. Each plate was divided into two halves such that 48 wells were serviced for TMD and 48 wells for OD from left to right. For each experiment, CCD841 cells were seeded in plates and incubated for 24 h. Following this incubation period, the media was removed, and the cells were treated with solutions of media containing increasing concentrations of dust (0, 10, 25, 50, 75, 100, 250, and 500 μg/mL) for an additional 24 h. It is important to note that the 0 μg/mL dust concentration serves as our sample/positive control, which refers to the expected outcome under normal conditions (i.e., cells without dust exposure). This is a common terminology in cell culture studies, where ‘positive control’ often refers to the normal or untreated cells, serving as a reference for the expected cell behavior in the absence of the experimental treatment. This control represents cells that were not exposed to dust and provides a baseline level of cellular activity against which the effects of dust exposure can be compared. This experimental design allows us to assess the impact of dust exposure on cell viability, cytotoxicity, and apoptosis in a controlled manner.

(a) MTT Assay Overview

MTT (3-(4,5-Dimethylthiazol-2-yl)-2,5-diphenyltetrazolium bromide) is a tetrazolium salt which is used to measure cellular metabolic activity as an indicator of cell viability and cytotoxicity [36]. Upon reduction, tetrazolium salts change into products that are deeply colored and can easily be measured using colorimetric methods. A purple formazan product is formed by MTT, which is solubilized prior to quantification [37].

For the MTT assay, CCD841 cells were seeded at a density of 5000 cells per well in 96-well plates and allowed to adhere overnight in a 37 °C incubator with 5% CO2. The following day, cells were exposed to various concentrations of dust samples (0, 10, 25, 50, 75, 100, 250, and 500 µg/mL) and incubated for another 24 h. Post-incubation, the medium was removed and 100 µL of MTT solution (5 mg/mL in PBS) was added to each well. After 4 h, the MTT solution was carefully discarded and 100 µL of DMSO was added to solubilize the formazan crystals. Absorbance was measured at 570 nm using a microplate reader.

(b) Protease Viability Assay Overview

The protease viability assay (GF-AFC assay) is a cell-permeable fluorogenic protease substance that is used to measure live-cell protease activity. It is worth noting that the GF-AFC assay is split by a live-cell protease to generate a proportional fluorescent signal, which is used to determine the number of cells that are viable [38]. This method has a shorter incubation time of 0.5–1 h as compared to that of tetrazolium assays of 1–4 h [39]. This approach gives room for multiplexing with other existing assays in similar sample cells, such as a bioluminescent assay, since it does not cause cells to lyse.

For the protease viability assay, CCD841 cells were plated at a density of 5000 cells per well in black clear-bottom 96-well plates. After overnight incubation, cells were treated with different dust sample concentrations (0, 10, 25, 50 h, 75, 100, 250, and 500 µg/mL) for 24 h. Post-incubation, 100 µL of the GF-AFC substrate was added to each well and incubated for 1 h at room temperature in the dark, and then fluorescence was measured using a fluorescence microplate reader.

(c) Cell Death by Apoptosis Assay

The Caspase-Glo 3/7 activation assay detects and quantifies cellular events that are associated with programmed cell death, including caspase activation, DNA fragmentation, and the exposure of the cell surface to phosphatidylserine (PS) [40]. Luminescent Caspase-Glo assays are used to identify the availability of caspases that are either involved in extrinsic or intrinsic apoptosis pathways.

For the Caspase-Glo 3/7 assay, CCD841 cells were seeded at a density of 5000 cells per well in white clear-bottom 96-well plates. Following overnight incubation, cells were treated with dust samples at different concentrations (0, 10, 25, 50, 75, 100, 250, and 500 µg/mL) for 24 h. After exposure, 100 µL of Caspase-Glo 3/7 reagent was added to each well and incubated at room temperature for 1 h. Luminescence was then measured using a luminometer.

(d) Cell Cytotoxicity Assay

Lactate dehydrogenase (LDH) is a soluble cytoplasmic enzyme present in almost all cells. It is released into the extracellular space when the plasma membrane is damaged, making it a reliable marker for cell damage and necrosis [37]. The softening of the plasma membrane is a crucial characteristic of necrotic cells, and the release of LDH can be measured in tissue culture conditions to quantify this phenomenon. This colorimetric assay provides a reliable means of determining cytotoxicity in both healthy and disease pathologies, where apoptosis and necrosis are two important mechanisms of cell death. Despite there being several methods for detecting apoptosis, there are just a handful for assessing necrosis, making LDH production a valuable tool for detecting necrosis when used in conjunction with other methods. For the LDH assay, CCD841 cells were plated at a density of 5000 cells per well in 96-well plates and allowed to adhere overnight. After incubation, cells were exposed to varying concentrations of dust samples (0, 10, 25, 50, 75, 100, 250, and 500 µg/mL) for 24 h. Following exposure, 50 µL of the cell culture supernatant was transferred to a new 96-well plate and mixed with 50 µL of the LDH reaction mixture, and then was incubated for 30 min at room temperature in the dark. Absorbance was then measured at 490 nm and 680 nm (background) using a microplate reader.

### 2.5. Statistical Analyses

Cell viability in percentages (%) was calculated as {[absorbance(sample) − absorbance (negative control)]/[(absorbance(sample control) − absorbance(negative control)]} × 100, where absorbance(sample), absorbance(negative control), and absorbance(sample control) are each average absorbances that were measured over three wells using a Synergy Mx microplate reader (BioTek, Winooski, VT, USA) to obtain the final absorbance reading. For visualization, we reported the fold change in cell viability with respect to the control/reference by dividing the % by 100. Cell death and cytotoxicity were reported in a similar fashion. In particular, the results are presented via bar graphs by averaging the fold change, with respect to the reference, from 4 independent experiments (n = 4), with error bars indicating the standard error of the fold change (StdErr). For each concentration, the calculations were performed in triplicate.

One and two-way analyses of variance (ANOVA) were carried out with SAS system software, version 9.4 (SAS Institute, Inc., Cary, NC, USA), where the treatment data at different concentrations were compared to control for each form of dust, and TMD was compared to OD, which was followed by a Tukey’s post-test. The level of significance was set at *p* < 0.05. The F values reported in the Results section are from these ANOVA tests. In ANOVA, the F statistic is a key part of determining whether our observed results could have happened by chance. The two numbers are the degrees of freedom associated with the statistical test. The first number is the degrees of freedom of the numerator (between-group variability) and the second number is the degrees of freedom of the denominator (within-group variability).

Generated overflow values which could not be measured with the plate reader were imputed by taking the median of the remaining replicates to maintain power while not influencing the effect size. Imputation occurred only for LDH-Glo: 1 time in plate 1 (1.04% of the 96 wells), 3 times in plate 2 (3.1%), 1 time in plate 3 (1.04%), and 2 times in plate 4 (2.1%).

## 3. Results

### 3.1. Cellular Metabolic Activity after Treatment with Indoor Dust Samples

**Figure 1:**
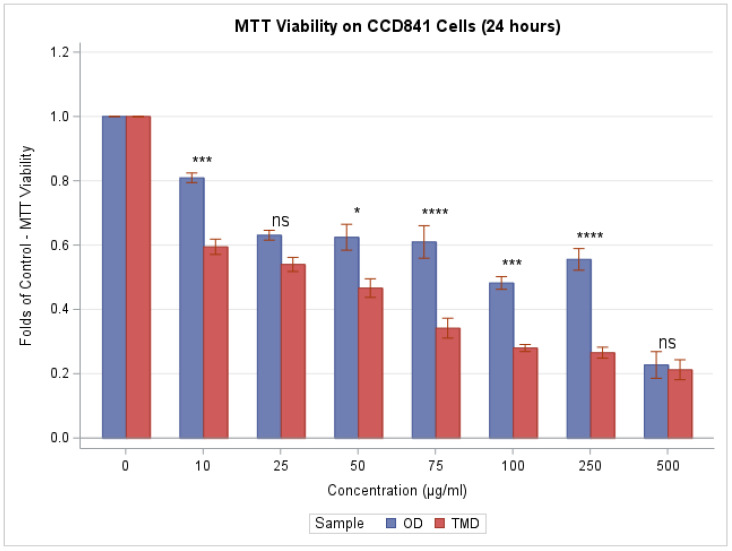
Cell viability: MTT assay. Higher concentrations of OD and TMD are associated with reduced cellular metabolic activity in CCD841 cells after 24 h of exposure. We use ns, *, **, ***, and **** to denote *p* > 0.05, *p* ≤ 0.05, *p* ≤ 0.01, *p* ≤ 0.001, and *p* ≤ 0.0001, respectively, when comparing TDM to OD via Tukey’s post hoc analysis of the two-way ANOVA.

Both dust samples (trace metal dust (TMD) and organic contaminant dust (OD)) substantially decrease cell viability in a concentration-dependent manner (F(7,24) = 124.69, *p* < 0.0001 for TMD; F(7,24) = 51.92, *p* < 0.0001 for OD), as shown in Figure 1, with TMD having a more pronounced effect (F(7,48) = 7.98, *p* < 0.0001). Folds of control were significantly lower for TMD than OD, and hence had a stronger effect, at the 10, 50, 75, 100, and 250 μg/mL concentrations (*p* = 0.0001, 0.0145, <0.0001, 0.0004, and <0.0001, respectively, according to Tukey’s post hoc analysis of the two-way ANOVA).

### 3.2. Triplex Protease

**Figure 2:**
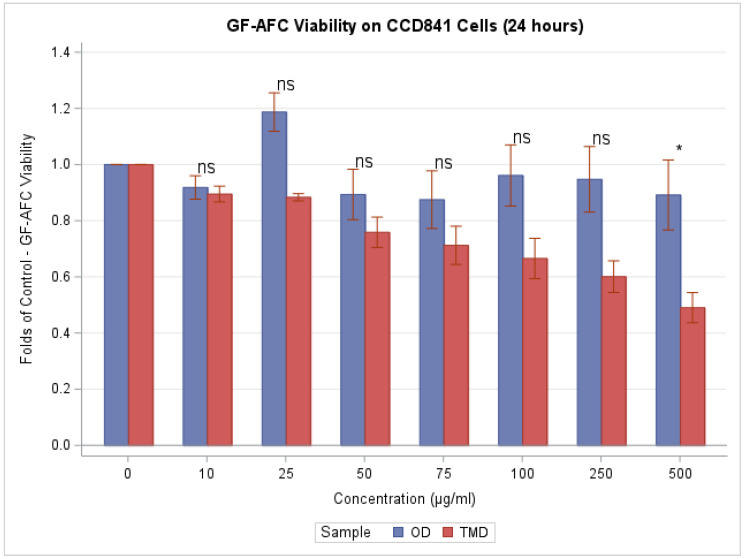
GF-AFC Viability Assay: At higher concentrations, cell protease activity decreases for TMD exposed cells. We use ‘ns’ to denote *p* > 0.05 and ‘*’ to denote *p* ≤ 0.05 when comparing TDM to OD via Tukey’s post hoc analysis of the 2-way ANOVA.

Figure 2 summarizes the results of protease analysis (GF-AFC). Our findings reveal that TMD significantly reduced colon cell viability in a concentration-dependent manner (F(7,24) = 11.56, *p* < 0.0001 for TMD; F(7,24) = 1.23, *p* = 0.3230 for OD). Differences in cell viability, with respect to control, between TMD and OD were marginally significant (F(7,48) = 2.11, *p* = 0.0605). Folds of control were significantly lower for TMD than OD, and hence had a stronger effect only at the 250 and 500 μg/mL concentrations (*p* = 0.0966, and 0.0245, respectively, according to Tukey’s post hoc analysis of the two-way ANOVA). At the highest concentrations of exposure (500 µg/mL), cell viability was decreased by 51.0 percent for TMD and 10.8 percent for OD relative to the control exposure.

### 3.3. Apoptosis Caspase 3/7:

**Figure 3:**
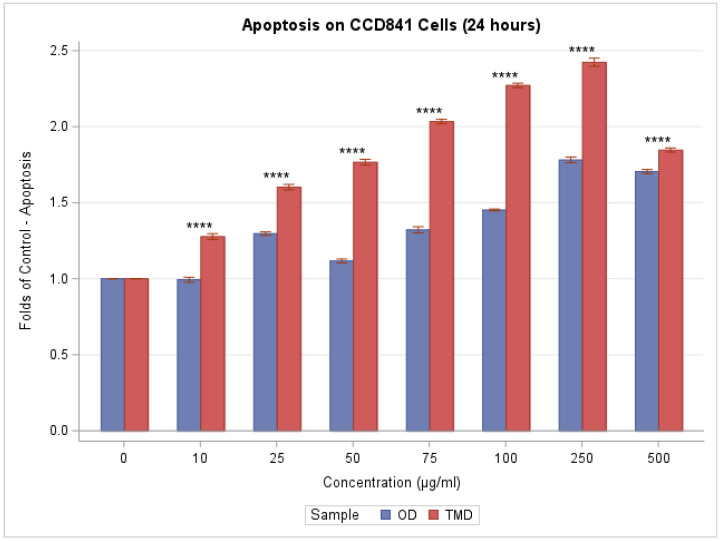
Caspase 3/7 apoptosis. At higher concentrations of OD and TMD, cell death increased. We use ns, *, **, ***, and **** to denote *p* > 0.05, *p* ≤ 0.05, *p* ≤ 0.01, *p* ≤ 0.001, and *p* ≤ 0.0001, respectively, when comparing TDM to OD via Tukey’s post hoc analysis of the two-way ANOVA.

The stimulation of caspase 3 and 7 was examined after 24 h of indoor dust exposure to investigate the process of CCD841 apoptosis. For TMD exposure, there was a monotonic, significant linear increase in apoptosis activity as concentrations increased until reaching the 250 µg/mL concentration (F(7,24) = 763.96, *p* < 0.0001). In comparison, OD dust exposure initially led to little change in apoptosis, but caspase activation was improved after reaching a concentration of approximately 25 µg/mL. Beginning at 25 µg/mL and beyond, apoptosis for OD was consistently significantly higher than at baseline, as shown in Figure 3 (F(7,24) = 455.54, *p* < 0.0001). The increase in apoptosis, measured in folds of control, for TMD was much more substantial than that of OD (F(7,48) = 179.63, *p* < 0.0001). Specifically, apoptosis, measured in folds of control, was significantly higher for TMD than for OD, and hence had a stronger effect at all concentrations (*p* < 0.0001 from Tukey’s post hoc analysis of the two-way ANOVA).

### 3.4. Cell Cytotoxicity in CCD841 after Treatment with Indoor Dust Samples (LDH Release)

**Figure 4:**
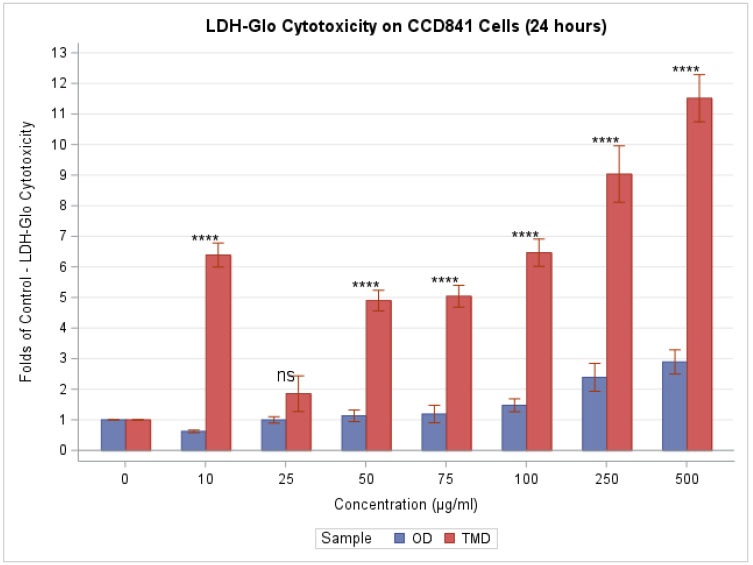
LDH-GLO cytotoxicity increased with higher concentrations, indicating that exposure to dust samples caused cellular membrane damage. We use ns, *, **, ***, and **** to denote *p* > 0.05, *p* ≤ 0.05, *p* ≤ 0.01, *p* ≤ 0.001, and *p* ≤ 0.0001, respectively, when comparing TDM to OD via Tukey’s post hoc analysis of the two-way ANOVA.

Following 24 h of exposure to dust, the amount of LDH produced by CCD841 cells was evaluated. An increase in the concentration of both forms of indoor dust produced demonstrable increases in LDH, as shown in Figure 4; however, there was a stronger effect for TMD (F(7,24) = 40.33, *p* < 0.0001 for TMD; F(7,24) = 8.94, *p* < 0.0001 for OD). In fact, the effect for OD was significant only at the 250 and 500 µg/mL concentration, as compared to the control, while that for TMD was significant at all concentrations except for 25 µg/mL. Further, except for the 25 µg/mL concentration, cell membrane damage, as quantified by LDH production, was demonstrably substantially higher for TMD compared to OD at all concentrations (*p* < 0.0001, 0.9861, <0.0001, <0.0001, <0.0001, <0.0001, and <0.0001, according to Tukey’s post hoc analysis of the two-way ANOVA). Treatments with 250 and 500 g/mL of dust greatly enhanced LDH release levels by up to 189% (i.e., 2.89 folds) for OD and 1152% (i.e., 11.5 folds) for TMD when compared to the untreated control.

## 4. Discussion

To the best of our knowledge, this study is one of the first to specifically investigate the effects of TMD and OD on cellular metabolic and protease activity, apoptosis, and biomarkers of cell membrane damage in CCD841 human colonic epithelial cells. Patterns appeared to be dose-dependent, with the highest TMD and OD exposures associated with the largest increases in apoptosis and LDH, as well as with the largest decrements in metabolic and protease activities. These findings further our understanding of the potential biological and health impacts of indoor dust exposure.

Cell viability and cytotoxicity were dramatically affected by indoor dust exposure. The MTT and protease assays clearly showed a more than 75% reduction in viable cells at high concentrations relative to the control cells. Importantly, dose–response relationships were also observed, with incrementally higher concentrations of TMD and OD cells seeing incrementally lower metabolic and protease activity. On the other hand, TMD and OD exposure led to obvious increases in LDH, suggesting that both exposures have detrimental impacts on colonic cell membrane integrity.

Apoptosis was also induced by indoor dust treatment, with the highest concentrations of TMD and OD increasing caspase 3 and 7 activation. Both indoor trace element and organic contaminates showed the elevation of caspase 3/7, starting from 10 µg/mL for TMD and 25 µg/mL for OD, and continued to increase with higher concentrations. Caspases 3 and 7 can influence numerous morphological and biochemical modifications during controlled extrinsic or intrinsic cell death, including the creation of apoptotic cells and DNA fragmentation [41]. Importantly, aberrant apoptosis may be an important determinant of a number of gastrointestinal diseases, including inflammatory bowel disease [42] and colon cancers [43].

Of note, the rise in the caspase 3/7 signal after exposure to TMD suggests that apoptosis is one mechanism contributing to cell death. However, the observed increase in LDH even at the lowest dose indicates that alterations to the cell membrane, potentially indicative of necrosis, are also likely to play a significant role in the cellular damage caused by TMD dust. Nevertheless, at higher TMD dosage treatments, namely 500 µg/mL, a drop in the caspase 3/7 signal was observed, along with an upsurge in the cytotoxicity indicator (LDH), indicating an increased number of dead cells and suggesting apoptosis via the necrotic mechanism. This reaction demonstrates that, in addition to chemical content, the dust treatment dosage impacts the type of cellular death process, as described by Nel et al. in 2001 [44]. These findings support the theory that the same substance can cause apoptosis at low quantities, but necrosis at larger concentrations [45]. Reduced diesel fume particles, for example, have been shown to cause cell apoptosis, whilst elevated doses have been shown to cause necrosis [44].

However, it is important to consider the context of these findings. First, the concentrations of dust used in our experiments, while representative of high-exposure scenarios, may not reflect the typical or average levels of dust that individuals are exposed to in their homes. The health effects of dust exposure can vary significantly depending on the concentration and type of dust particle, as well as the duration of exposure [46,47]. In real-world scenarios, individuals are often exposed to lower concentrations of dust over prolonged periods, which can have different health effects than short-term, high-concentration exposure [48,49,50].

Second, while our study provides valuable insights into the potential health effects of TMD and OD, it is important to note that indoor dust is a complex mixture of various substances, and the composition can vary widely depending on the source and location [51,52,53]. Therefore, the health effects of dust exposure can also vary depending on the specific composition of the dust. In our study, we used standard samples of TMD and OD, which are representative of typical indoor dust contaminants. However, the specific health effects of dust exposure may vary depending on the presence and concentrations of other contaminants not included in these standard samples [54].

Third, our study focused on the effects of dust exposure on CCD841 human colonic epithelial cells. While these cells are a useful model for studying the effects of dust ingestion, they may not fully represent the complex interactions that occur in the human gastrointestinal tract during dust ingestion. Future studies could benefit from using more complex models, such as co-cultures of different cell types, to better simulate the conditions in the human gastrointestinal tract [54].

Despite these considerations, our findings contribute to the growing body of literature on the potential biological and health impacts of indoor dust exposure. They highlight the need for further research to better understand the health risks associated with indoor dust exposure, particularly in relation to gastrointestinal health. Our study, like all in vitro studies, has several important limitations. First, while we identified viability, cell death, and cytotoxicity in CCD 841 CoN cell cultures, which are of female embryonic origin (21 weeks gestation), these findings may not fully represent the diverse responses that could be observed in a human population with varying characteristics such as sex and age. In vitro studies are inherently limited in their ability to replicate the complexity of biological systems in living organisms. Another limitation to our study is the lack of comparative data with known inducers of viability, apoptosis, and cytotoxicity. A further limitation of our study is that we only investigated the effects of a 24 h exposure period. In real-world scenarios, exposure to indoor dust can be continuous and long-term. Future studies could investigate the effects of different exposure durations to better simulate real-world conditions.

While our study provides valuable insights into the potential effects of indoor dust exposure on human colonic cells, it is important to note that the doses tested in our study may not directly correspond to the amounts of dust ingested by children in a real-world setting. The experimental design of our study was intended to provide a preliminary understanding of the potential effects of dust ingestion on human colonic cells, rather than to simulate the exact conditions of dust exposure in humans. The doses used in our study were intended to represent a range of potential exposure levels, and the effects observed at the higher doses may not necessarily occur under typical exposure conditions. The effects of long-term, low-dose exposure to indoor dust are still not fully understood and warrant further investigation.

## 5. Conclusions

In this study, we provided novel insights into the effects of TMD and OD on human colonic cells (CCD841). Our experimental data revealed that exposure to TMD and OD significantly decreased cellular metabolic and protease activity, with a decrease in cell viability of up to 51.0 percent for TMD and 10.8 percent for OD at the highest exposure concentration (500 µg/mL) compared to the control. Additionally, we observed an increase in apoptosis and biomarkers of cell membrane damage (LDH), indicating potential cytotoxic effects of indoor dust exposure. These findings contribute to the growing body of knowledge on the potential biological and health impacts of indoor dust exposure, particularly in the context of gastrointestinal diseases. However, it is important to note that the doses tested in our study may not directly correspond to the amounts of dust ingested in a real-world setting. The effects of long-term, low-dose exposure to indoor dust are still not fully understood and warrant further investigation. Future research should continue to explore these physiological mechanisms to further our understanding and inform public health interventions.

## Data Availability

The data presented in this study are available on request from the first author. The data are not publicly available to maintain the integrity of its use.
